# TDP-43 Oligomerization and Phase Separation Properties Are Necessary for Autoregulation

**DOI:** 10.3389/fnins.2022.818655

**Published:** 2022-04-14

**Authors:** Lydia C. Koehler, Zachary R. Grese, Alliny C. S. Bastos, Lohany D. Mamede, Tomasz Heyduk, Yuna M. Ayala

**Affiliations:** Edward Doisy Department of Biochemistry and Molecular Biology, Saint Louis University, St. Louis, MO, United States

**Keywords:** TDP-43 (TAR DNA-binding protein 43), RNA binding protein, ALS, frontotemporal dementia (FTD), liquid-liquid phase separation (LLPS), protein aggregation, TDP-43 autoregulation, ALS mutations

## Abstract

Loss of TDP-43 protein homeostasis and dysfunction, in particular TDP-43 aggregation, are tied to amyotrophic lateral sclerosis (ALS) and frontotemporal dementia (FTD). TDP-43 is an RNA binding protein tightly controlling its own expression levels through a negative feedback loop, involving TDP-43 recruitment to the 3′ untranslated region of its own transcript. Aberrant TDP-43 expression caused by autoregulation defects are linked to TDP-43 pathology. Therefore, interactions between TDP-43 and its own transcript are crucial to prevent TDP-43 aggregation and loss of function. However, the mechanisms that mediate this interaction remain ill-defined. We find that a central RNA sequence in the 3′ UTR, which mediates TDP-43 autoregulation, increases the liquid properties of TDP-43 phase separation. Furthermore, binding to this RNA sequence induces TDP-43 condensation in human cell lysates, suggesting that this interaction promotes TDP-43 self-assembly into dynamic ribonucleoprotein granules. In agreement with these findings, our experiments show that TDP-43 oligomerization and phase separation, mediated by the amino and carboxy-terminal domains, respectively, are essential for TDP-43 autoregulation. According to our additional observations, CLIP34-associated phase separation and autoregulation may be efficiently controlled by phosphorylation of the N-terminal domain. Importantly, we find that specific ALS-associated TDP-43 mutations, mainly M337V, and a shortened TDP-43 isoform recently tied to motor neuron toxicity in ALS, disrupt the liquid properties of TDP-43-RNA condensates as well as autoregulatory function. In addition, we find that M337V decreases the cellular clearance of TDP-43 and other RNA binding proteins associated with ALS/FTD. These observations suggest that loss of liquid properties in M337V condensates strongly affects protein homeostasis. Together, this work provides evidence for the central role of TDP-43 oligomerization and liquid-liquid phase separation linked to RNA binding in autoregulation. These mechanisms may be impaired by TDP-43 disease variants and controlled by specific cellular signaling.

## Introduction

**FIGURE 1:**
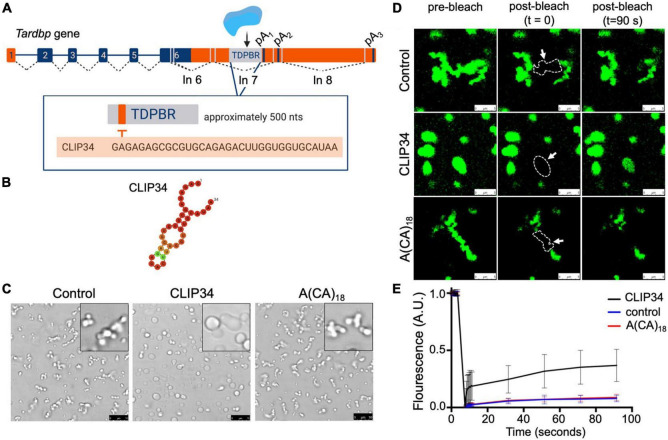
Binding to the RNA region mediating TDP-43 autoregulation increases the liquid properties of TDP-43 condensates. **(A)** *Tardbp* pre-mRNA organization into introns (lines) and exons (boxes), highlighting coding regions (blue boxes). Splicing is denoted by dashed lines and the approximate positions of alternative splicing in the 3′ UTR are shown (gray lines). TDP-43 binds to its cognate mRNA transcript in the 3′ UTR TDP-43 binding region (TDPBR, gray box), including the 34-nt sequence CLIP34. **(B)** Lowest free energy secondary structure of CLIP34 predicted by RNAfold. The calculated frequency of this structure in the ensemble is 53%. **(C)** Condensates formed by purified TDP-43 (4 μM) as seen by brightfield microscopy without RNA (control), in the presence of CLIP34 RNA (4 μM), or the non-specific control RNA A(CA)_18_ (4 μM) at 150 mM NaCl, pH 7.5. Representative images of 3 independent experiments from 2 protein preparations. Scale bars, 10 μm. **(D)** Fluorescence recovery after photobleaching (FRAP) of condensates (white arrow) formed by Oregon green labeled-TDP-43 (10% of total protein) mixed with CLIP34, A(CA)_18_, or in the absence of RNA in the same experimental conditions as **(C)**. Dotted line and arrow define the region of interest. Scale bars, 5 μm. **(E)** Quantification of fluorescence recovery in panel **(D)**, showing mean values and SD from 3 biological replicates consisting of 16 measurements, performed from 2 different protein purifications.

Aggregation, cellular mislocalization and loss of nuclear TDP-43 (TAR DNA binding protein) are hallmarks of amyotrophic lateral sclerosis (ALS), frontotemporal dementia (FTD) (Arai et al., 2006; Neumann et al., 2006) and limbic-predominant age-related TDP-43 encephalopathy (LATE) (Nelson et al., 2019). In addition, TDP-43 inclusions are associated with multisystem proteinopathy (MSP) (Weihl et al., 2009) and other neurodegenerative disorders, including Alzheimer’s disease (AD) and chronic traumatic encephalopathy (CTE) (McKee et al., 2010). TDP-43 is an RNA binding protein whose cellular levels are tightly controlled as its overexpression leads to increased cytoplasmic accumulation and aggregation. In addition, even moderate changes in TDP-43 protein expression disrupt the regulation of target genes (Arnold et al., 2013). Abnormal increase in TDP-43 levels results in neurotoxicity as observed in human neurons (Barmada et al., 2010) and in a wide range of animal models, including non-human primates (Tatom et al., 2009; Wils et al., 2010; Xu et al., 2010; Estes et al., 2011; Uchida et al., 2012; Diaper et al., 2013). So far, TDP-43 autoregulation through a negative feedback loop is the only known mechanism controlling TDP-43 expression levels. During this process, TDP-43 binds to its own mRNA 3′UTR and inhibits protein synthesis (Ayala et al., 2011; Polymenidou et al., 2011; Figure 1A). This autoregulatory mechanism, reviewed in Tziortzouda et al. (2021), is conserved among vertebrates and its disruption in mouse models results in widespread splicing changes, neurotoxicity (Xu et al., 2010; Arnold et al., 2013; Sugai et al., 2019) and increased cytoplasmic TDP-43 aggregation (D’Alton et al., 2015; Koyama et al., 2016). The existence of mutations that are causative of ALS in noncoding regions involved in autoregulation (Gitcho et al., 2009; Pesiridis et al., 2009) and the finding that one of these mutations shows increased TDP-43 transcript expression (Gitcho et al., 2009), strongly suggest that defects in self-regulation may result in disease. This is also supported by a knock-in mouse model in which the Q331K ALS mutation exhibits cognitive impairment and disrupts autoregulation (White et al., 2018). Furthermore, accumulation of misfolded TDP-43 aggregates potentially increases protein production in affected cells by triggering a feed-forward mechanism, in which TDP-43 is sequestered and is no longer able to self-regulate, as suggested by recent studies (Sugai et al., 2019). In agreement with this model, ALS-affected motor neurons and FTD neurons that lack nuclear TDP-43 show impaired *Tardbp* autoregulation (Koyama et al., 2016; Liu et al., 2019). Collectively, these findings indicate that *Tardbp* may be one of the most critical TDP-43 targets to prevent the loss of protein homeostasis and neurotoxicity. Therefore, improved understanding of the factors controlling TDP-43 autoregulation are necessary to test this model and provide new avenues to maintain TDP-43 proteostasis. However, despite the central importance of this process in TDP-43 function and its link to disease, the mechanisms involved in TDP-43 autoregulation are ill-defined, and the factors controlling autoregulation are even less well understood.

The cellular organization of RNA binding proteins, which in turn impacts protein function, involves condensation into ribonucleoprotein (RNP) granules. This process is mediated by liquid-liquid phase separation (LLPS) into often dynamic, liquid-like complexes (Brangwynne et al., 2009; Wippich et al., 2013; Feric et al., 2016). The high concentration of the components while retaining dynamic properties within the droplets is fundamental for RNP granule function and organization in cells [reviewed in Alberti et al. (2019)]. Formation of RNP granules is mediated by multivalent interactions, including RNA binding and self-assembly through different protein domains, such as low complexity regions and oligomerization domains (Li et al., 2012; Lin et al., 2015; Zhang et al., 2015; Garcia-Jove Navarro et al., 2019). The liquid properties of these assemblies and their multivalent properties enable rapid regulation in response to cellular stimuli [reviewed in Shin and Brangwynne (2017); Wang et al. (2021)]. RNA is a principal component of RNP granules and recent studies, including our own, suggest that RNA binding modulates the LLPS properties of TDP-43 through specific interactions (Zhang et al., 2015; Garcia-Jove Navarro et al., 2019; Grese et al., 2021). However, how LLPS affects RNA processing following recruitment to the transcripts, and whether this is a gene-dependent mechanism is currently not known. Importantly, numerous studies also suggest that defects in LLPS are associated with disease, such as in the case of TDP-43, FUS (Fused in sarcoma) and other RNA binding proteins linked to neurodegeneration (Lin et al., 2015; Molliex et al., 2015; Patel et al., 2015). Specifically, conversion of the condensates into fibrils or complexes with solid-like properties is thought to cause accumulation of protein aggregates associated with pathology. Therefore, further insight on the regulation of TDP-43 LLPS properties and their involvement in controlling the expression of TDP-43 targets will help elucidate important pathogenic factors.

We sought to define the molecular mechanisms in TDP-43 autoregulation and determine the effect disease-associated conditions have in this process. Our results strongly suggest that autoregulation is mediated by TDP-43 self-assembly and LLPS upon RNA binding. In addition, specific TDP-43 binding to RNA sequences that mediate autoregulation promotes liquid properties of TDP-43 condensates or liquid droplets. In addition, we find that TDP-43 posttranslational modifications and disease-associated variants may alter autoregulation by modulating TDP-43 condensation. Together, these findings highlight the potential of developing therapies based on controlling pathways linked to TDP-43 autoregulation as well as specific TDP-43-RNA binding interactions.

## Materials and Methods

### Reagents and Chemicals Were Purchased From Sigma-Aldrich Unless Otherwise Specified

#### Plasmid Construction

The HA tagged TDP-43 sequence was cloned into pcDNA5™/FRT/TO (Thermo Fisher) between BamHI and NotI restriction sites. Quikchange- Site directed mutagenesis protocol (Agilent) was performed to create single-site and multiple-site mutations. The ΔCR deletion missing a.a. 316–346 was generated from gBlock sequence. pcDNA5 HA-mEGFP-TDP constructs were created by subcloning TDP-43 cDNA into the mEGFP-C1 vector (Addgene) between XhoI and HindIII. HA-mEGFP-TDP-43 was subcloned into pcDNA5™/FRT/TO using EcoRV and NotI. Oligonucleotides and gBlock sequence are listed in the Supplementary Table 1.

#### Expression and Purification of Recombinant TDP-43

Recombinant TDP-43 expression and purification was carried out as previously described (Grese et al., 2021).

#### Liquid-Liquid Phase Separation, Turbidity Assays, and Fluorescence Recovery After Photobleaching

The protocols developed in our lab for these assays were recently described (Grese et al., 2021). Microscopy images were taken following equilibration of condensates onto glass coverslips, approximately 60 min after the starting point of the reaction. For turbidity assays, data points were taken as the absorbance at 600 nm λ at 25 min. Data points were then normalized using the equation *Abs*_*norm*_=(*Abs*−*Abs*_*blank*_)/(*Abs*_*max*_−*Abs*_*blank*_), where *Abs*_*max*_ is the condition being normalized to 1. The RNA oligonucleotides used in these experiments are listed in Supplementary Table 3. FRAP was performed as previously described (Grese et al., 2021).

#### Fluorescence Anisotropy

Purified TDP-43 WT and mutants previously purified were serially diluted in a 1:2 ratio in a final range of 0–2 μM protein into a 300 mM NaCl, 10 mM Tris (pH 8.0), 5% glycerol, 5% sucrose, 0.5 mM TCEP buffer solution. The protein dilution was mixed with CLIP34 (IDT) 3′ labeled with FITC (fluorescein isothiocyanate) in a final concentration of 100 nM and added in triplicate in a 384-well black flat bottom plate (Corning) in a total reaction volume of 30 μL protected from light. The anisotropy measurements were performed in a Spectra Max i3 plate reader (Molecular Devices) with excitation and emission wavelengths of 480 and 520 nm, respectively. To assess the binding, the anisotropy data was fitted in a nonlinear fit in 4-parameter logistic model to calculate the apparent IC_50,*app*_. The apparent IC_50_ (IC_50,*app*_) is the protein concentration in which 50% of the maximal anisotropy change is observed. Data analysis were performed using GraphPad Prism 9.

#### Cell Culture and Stable Cell Line Production

HEK293 Flp-In™ T-REX™ cells (Thermo Fisher) were maintained in DMEM (Dulbecco’s Modified Eagle’s Medium—high glucose, Sigma) with 10% FBS (fetal bovine serum) and incubated at 37°C and 5% CO_2_. Stable cell lines were achieved through co-transfection of pcDNA5™/FRT/TO/HA or mEGFP constructs and pOG44 using Lipofectamine 3000™ (Invitrogen). Cells were selected using 75 μg/mL hygromycin (Sigma).

#### Cell Collection, Lysis, and Induction of LLPS Using Cell Lysates

This protocol was modified from Freibaum et al. (2021) and described in Grese et al. (2021). RNA oligonucleotides for these assays (Supplementary Table 3) were synthesized by Integrated DNA Technologies. In addition, oligonucleotides modified with a 2′OMe group and phosphorothioate backbone were used in the experiments carried out at 250 mM NaCl.

#### Cycloheximide Post-Chase Experiment

WT and M337V HA-TDP stable cells were plated and induced with 1 μg/mL of tetracycline for 72 h to reach confluency of 85–90%. Media was then changed to DMEM, 10% FBS, 100 μg/mL cycloheximide. Samples were taken at 0, 4, 8, 16, 32, and 40 h. Each sample was washed once with PBS and collected by centrifugation. Cell pellets were immediately lysed in cell lysis buffer and sonicated before being prepared for immunoblotting.

#### Cell Lysate RIPA/Urea Fractionation

WT and M337V stable cell lines were plated and induced for 72 h before being harvested by centrifugation. Cell pellets were then resuspended in RIPA Buffer [50 mM Tris pH 8.0, 150 mM NaCl, 1% NP-40, 5 mM EDTA, 0.5% SOC, 0.1% SDS, 1x protease/phosphatase inhibitor (SigmaFAST EDTA-free protease inhibitor cocktail tablet)] and sonicated. Total fraction samples were taken after sonication and the samples were then centrifuged at 98,400 *g* for 30 min at 4°C. The pellet was then rinsed with RIPA buffer and centrifuged again. The resulting pellet was resuspended in urea buffer (7 M urea, 2 M thiourea, 4% CHAPS, 30 mM Tris pH 8.5). Fractions were quantified by western blot as described above.

#### Immunoblotting

Immunoblotting was carried out using standard western blotting techniques and the antibodies for TDP-43 (Proteintech, 10782-2-AP), FUS/TLS (Santa Cruz Biotechnologies, sc47711), matrin-3 (Abcam, ab151739), and GAPDH (Abcam, Cat. No. ab181602). Membranes were imaged using Odyssey scanning (LI-COR) and quantified using ImageStudioLite software (LI-COR).

#### RNA Extraction and Quantitative RT-PCR

Trizol Reagent (Thermo Fisher) was used for RNA extraction and was carried out according to manufacturer instructions. M-MLV reverse Transcriptase (Thermo Fisher) was used for cDNA production according to the manufacturer instructions. Each primer mentioned in Supplementary Table 2 was used at a final concentration of 0.4 μM for a CFX96 Real-Time PCR detection system.

## Results

### Specific TDP-43 Binding to an RNA Sequence Linked to Autoregulation Increases the Liquid Properties of TDP-43 Condensates

TDP-43 binds to an extended region in the 3′ UTR of its own transcript (*Tardbp*), spanning approximately 500 nucleotides, during autoregulation (Figure 1A; Ayala et al., 2011; Polymenidou et al., 2011; Tollervey et al., 2011; Bhardwaj et al., 2013). This TDP-43 binding region (TDPBR) is immediately upstream of the proximal alternative polyadenylation site pA_1_ and is included in an alternatively spliced intron (in7). The mechanisms proposed to follow TDP-43 recruitment to TDPBR are based on analyses of the endogenous *Tardbp* transcript and derived gene reporters. These studies support the engagement of different processes during autoregulation: alternative polyadenylation, alternative splicing producing isoforms targeted for degradation *via* nonsense mediated decay (NMD), and nuclear retention of specific transcripts. Detailed studies by Koyama et al. (2016) suggest that autoregulation results from a combination of these mechanisms, but the extent to which each of these pathways contributes to the control of TDP-43 expression remains unknown (Polymenidou et al., 2011; Bembich et al., 2014; Koyama et al., 2016). A 34-nt. region within TDPBR (CLIP34) is commonly found in cross-linking immunoprecipitation (CLIP) analyses of TDP-43-bound transcripts in human cells and mouse brain (Polymenidou et al., 2011; Tollervey et al., 2011; Figures 1A,B). This isolated sequence is capable of decreasing TDP-43 toxicity in neurons, suggesting that CLIP34 binding prevents TDP-43 aggregation and aberrant phase transitions (Mann et al., 2019). We recently reported that CLIP34 was among the GU-rich RNA sequences capable of modulating TDP-43 phase separation properties (Grese et al., 2021). In the present work, we sought to gain further insight of the mechanisms that mediate TDP-43 LLPS linked to CLIP34 and explore whether LLPS plays a role in TDP-43 autoregulatory function. For this, TDP-43 LLPS was analyzed using purified full-length TDP-43 and we observed a dramatic change in the behavior of TDP-43 condensates in the presence of CLIP34. At 150 mM NaCl in the absence of RNA, TDP-43 forms small condensates that fail to coalesce and cluster into chain-like structures (Figure 1C). As previously shown, these rapidly obtain fibril-like properties that are more similar to gel or solid complexes instead of behaving as liquid droplets (French et al., 2019). Remarkably, addition of CLIP34 promoted TDP-43 condensate fusion and coalescence increasing droplet size. This was not observed in the presence of an RNA oligonucleotide control sequence of similar length, A(CA)_18_. We measured the size of the condensates in the different conditions to quantify the changes. The condensates mediated by CLIP34 are more than twofold significantly larger than control conditions (Supplementary Figure 1). Addition of A(CA)_18_ showed no significant difference relative to control. These observations are in agreement with recently published data from our lab, showing that CLIP34 promotes TDP-43 condensation at higher NaCl concentration (250 mM). Our findings suggest that at physiological salt conditions (150 mM NaCl) TDP-43 binding to CLIP34 specifically increases the liquid and dynamic properties of TDP-43 condensates. Fluorescence recovery after photobleaching (FRAP) of the TDP-43-CLIP34 condensates showed rapid recovery of Oregon green-labeled TDP-43 indicating a dynamic exchange between the light and dense phases. We observed approximately 40% recovery in TDP-43 condensates formed with specific-binding RNA (CLIP34), while condensates without RNA or in the presence of non-binding RNA showed virtually no recovery (Figures 1D,E). This suggests that while a substantial immobile fraction is present in the TDP-43-CLIP34 sample, there is a difference between the dynamics of the specific vs. non-specific RNA conditions. The immobile fraction present in the CLIP34 condition could be explained by the tight binding between TDP-43 and CLIP34 RNA, as the recovery could be partially dependent on exchange of RNA-bound and free labeled TDP-43, and thereby the off-rate of the TDP-43-RNA interaction. TDP-43-CLIP34 binding affinity is in the nanomolar range suggesting that the off-rate may be slow and this could explain the slow fluorescence recovery. In addition, the amount of CLIP34 in our reaction may also affect the degree of recovery. High RNA concentrations in similar experiments causing FRAP values that do not recover to baseline levels have been previously observed (Zhang et al., 2015). Our results are further supported by videos of condensates that show the dynamic properties of these complexes. Here, we show the marked effect of CLIP34, which increased condensate fusion and coalescence, generating larger complexes compared to control and A(CA)_18_ (Supplementary Movies 1–3). Collectively, our findings indicate that TDP-43 phase separation may be modulated by binding to CLIP34 specifically, preventing fibrillization of TDP-43 condensates.

**FIGURE 2:**
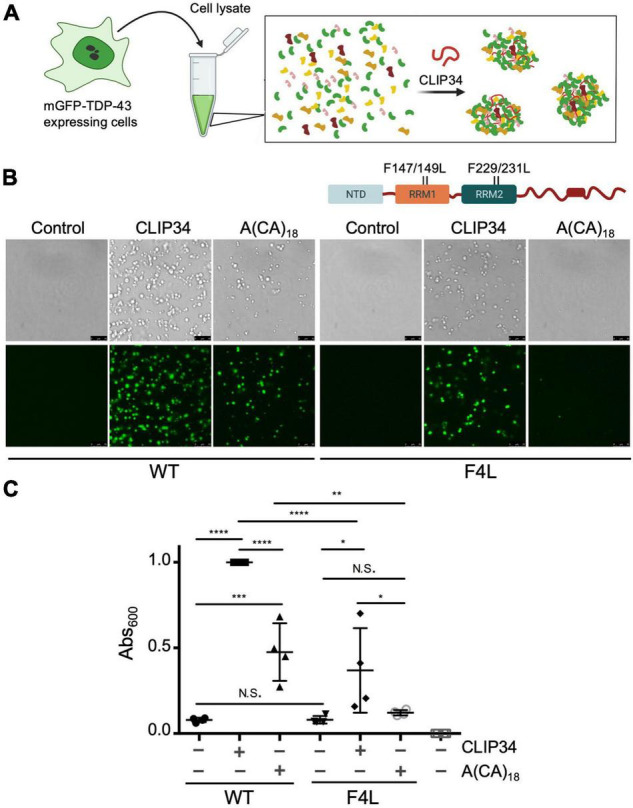
CLIP34 RNA promotes TDP-43 condensation in a cellular lysate. **(A)** Cell lysates prepared from HEK293 cells stably expressing a single mGFP-tagged copy of either wild-type (WT) or F147/149/229/231L (F4L) TDP-43. Recombinant TDP-43 (WT or F4L, 5.3 μM) was added to the cell lysate at 250 mM NaCl, pH 7.5. Samples were mixed with CLIP34 (7.6 μM), A(CA)_18_ (7.6 μM), or no RNA control. **(B)** Condensates were imaged by brightfield and fluorescence microscopy. Representative images of 3 biological replicates from 2 protein preparations. Scale bars, 10 μm. **(C)** Phase separation of TDP-43 in cell lysates measured by turbidity in the same experimental conditions as panel B. Mean and SD of 4 biological replicates from 2 recombinant protein preparations. Analyzed by one-way ANOVA [*F*(6,21) = 38.51, *P* < 0.0001]. Sidak’s multiple comparisons test was used to compare selected groups. *P ≤ 0.05 **P ≤ 0.01, *** *P* ≤ 0.001, ****P ≤ 0.0001, N.S., no significance. The last column corresponds to blank, buffer only sample.

To test whether condensation of CLIP34-bound TDP-43 may be observed in a cellular environment, we adapted a recently developed protocol to observe biomolecular condensates in human cellular lysate (Freibaum et al., 2021). We prepared cellular extract from human embryonic kidney cells (HEK293) cells stably expressing a single copy of monomeric mEGFP-TDP-43 wild-type (WT) (Figure 2A). The lysate was mixed with purified TDP-43 after addition of RNA. At 250 mM NaCl, in the absence of RNA, the mixture appeared homogeneous and lacked visible foci (Figure 2B). However, addition of CLIP34 readily promoted formation of mEGFP-TDP-43-positive granules. Control conditions in which no CLIP34 was added did not promote mEGFP-TDP-43 condensation. As a control, we tested the effect of A(CA)_18_ RNA and observed a marked decrease in phase separation compared to CLIP34. The condensates observed with A(CA)_18_, which is not expected to bind TDP-43 specifically (Buratti and Baralle, 2001), may be due to interactions of cell lysate components with the oligonucleotide and TDP-43 through non-specific binding. Additionally, the higher concentration of TDP-43 and RNA that was required to generate condensate formation under these conditions could promote liquid-liquid phase separation through less specific interactions. We also found that, at lower salt concentrations (150 mM NaCl), purified TDP-43 and cell lysate resulted in the formation of the small condensates clustering together into fibril-like structures (Supplementary Figure 2). However, addition of CLIP34 generated larger round-shaped condensates, suggesting increased liquid properties consistent with our experiments using purified TDP-43 alone. This was not observed upon addition of A(CA)_18_ RNA (Supplementary Figure 2B). To further test the requirement of specific TDP-43 binding to CLIP34, we analyzed RNP granule formation of cellular lysate from HEK293 cells expressing an RNA binding-deficient mutant mEGFP-TDP-43 F4L (Phe147/149/229/231Leu) (Buratti and Baralle, 2001). Addition of CLIP34 to the F4L cellular lysate showed a decrease in condensate formation relative to WT (Figure 2B). We also observed a dramatic decrease in the in the number and size of granules formed in F4L cell lysates mixed with A(CA)_18_, relative to WT. To further compare and quantify TDP-43 condensation in the various conditions, we measured lysate turbidity, as this often correlates with phase separation (Babinchak and Surewicz, 2020; Grese et al., 2021). Addition of CLIP34 increased the turbidity more than threefold compared to lysate or buffer only controls (Figure 2C). Lysate turbidity upon mixing with CLIP34 was also significantly higher than A(CA)_18_-mixed lysate, consistent with our imaging data. Addition of CLIP34 to F4L cellular lysate showed 50% reduction in turbidity compared to WT. As the imaging studies showed, addition of A(CA)_18_ to F4L lysate did not significantly increase turbidity compared to control. This data suggests that in the context of cellular lysate, the remaining TDP-43-RNA contacts of F4L are sufficient for condensate formation, albeit with lower efficiency relative to WT. Collectively, these results indicate that CLIP34 binding specifically increases the liquid properties of TDP-43 condensates and promotes phase separation of TDP-43 in cell-like conditions.

### TDP-43 Autoregulation Is Mediated by Oligomerization and Phase Separation

**FIGURE 3:**
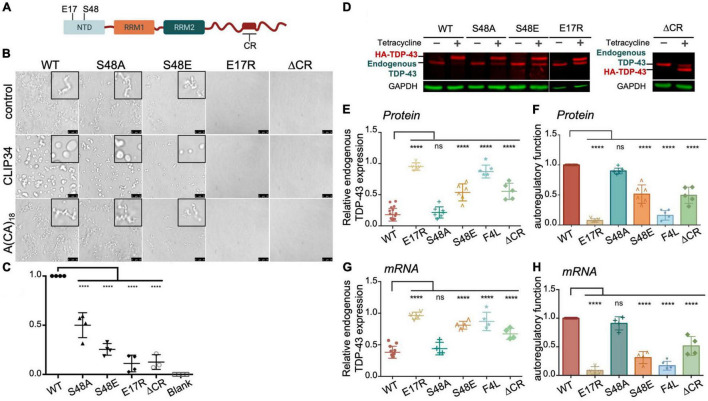
TDP-43 autoregulation in human cells depends on protein self-assembly and phase separation. **(A)** Single site amino acid substitutions at E17 and S48 in the N-terminal domain of TDP-43 and deletion of the highly conserved region (CR) in the C-terminal domain are indicated. **(B)** Phase separation of purified WT and mutant TDP-43 (4.8 μM) as seen by brightfield microscopy without RNA (control), in the presence of CLIP34 RNA (4.8 μM), or the non-specific control RNA A(CA)_18_ (4.8 μM) at 150 mM NaCl, pH 7.5. Representative images of three biological replicates from 2 protein preparations. Scale bars, 10 μm. **(C)** Phase separation of WT and mutant TDP-43 measured by turbidity in the same experimental conditions as panel **(B)**. Mean and SD of at least three biological replicates from 2 recombinant protein preparations. Analyzed by one-way ANOVA [*F*(4,14) = 81.45, *P* < 0.0001]. Dunnett’s multiple comparisons test was used to compare groups to WT. ****P ≤ 0.0001. The last column corresponds to blank, buffer only sample. **(D)** Representative immunoblots of HEK293 cell lines stably expressing HA-tagged TDP-43 upon tetracycline induction for 72 h. Transgene and endogenous TDP-43 signals are indicated. Immunoblots were probed with TDP-43 antibody (red) and GAPDH as loading control (green). **(E)** Reduction of endogenous TDP-43 protein levels upon expression of the transgene quantified from panel **(D)**. Cells expressing the RNA binding-deficient mutant F4L were used for comparison. **(F)** Autoregulatory activity of each mutant, relative to WT, calculated from values in panel **(E)** as endogenous TDP-43*^WT^*/endogenous TDP-43*^mutant^*. **(G,H)** Autoregulation in terms of *Tardbp* transcript levels measured by qPCR and quantified as in panels **(E,F)**. Mean and SD of ≥ 5 and four biological replicates for protein and RNA, respectively. Analyzed by one-way ANOVA, **(E)** [*F*(10,69) = 111.6, *P* < 0.0001], **(F)** [*F*(11,58) = 75.02, *P* < 0.0001], **(G)** [*F*(10,43) = 54.64, *P* < 0.0001] and **(H)** [*F*(9,37) = 97.40, *P* < 0.0001]. Dunnett’s multiple comparisons test was used to compare selected groups. ****P ≤ 0.0001.

Based on our results showing modulation of TDP-43 phase separation by CLIP34, we next asked whether LLPS plays a role during TDP-43 autoregulation in human cells. For this, we introduced site directed mutations in different TDP-43 domains that disrupt self-assembly and phase separation (Figure 3A). We targeted the N-terminal domain (NTD) with specific substitutions that inhibit oligomerization. Residue E17 of each NTD monomer makes multiple contacts upon dimerization, whereby the N-terminal domains are arranged in head-to-tail configuration (Afroz et al., 2017; Wang et al., 2018). Consequently, the E17R mutation greatly impairs NTD oligomerization. We also evaluated the role of S48 in the NTD, which undergoes phosphorylation in human cells (Rigbolt et al., 2011; Hornbeck et al., 2015; Wang et al., 2018). The phosphomimetic substitution S48E disrupts oligomerization and LLPS of full-length TDP-43 (Wang et al., 2018), suggesting that S48 phosphorylation modulates TDP-43 self-assembly. In agreement with these previous results, we observed fewer S48E condensates in the presence or absence of CLIP34 compared to WT or to the phospho-null substitution S48A (Figure 3A). At the same time, S48A showed moderately decreased condensation compared to WT under control and CLIP34 conditions. We found that in stark contrast with WT TDP-43, E17R failed to phase separate and addition of CLIP34 was unable to promote condensation of this mutant (Figure 3B). As further validation of the role of LLPS in CLIP34-mediated condensates, we deleted an evolutionarily conserved region in the C-terminal domain (a.a. 316-343) (ΔCR) that forms a partially α-helical structure, which is important for self-assembly and phase separation (Conicella et al., 2016, 2020; Schmidt and Rohatgi, 2016). As seen with the NTD oligomerization mutants, ΔCR inhibited LLPS in the presence or absence of RNA (Figure 3B). To better compare the differences in LLPS among the variants, we quantified phase separation in the presence of CLIP34 by turbidity (Figure 3C). Compared to WT, disruption of NTD-mediated oligomerization by E17R and S48E showed greater than 80 and 70% significant reduction in turbidity, respectively. Consistent with the microscopy data, S48A reduced turbidity compared to WT, but not as much as S48E. The ΔCR mutant showed greater than 80% significant reduction in turbidity. We examined whether the defects in CLIP34-driven LLPS were caused by decreased mutant binding to the RNA, quantifying the TDP-43-CLIP34 interactions by fluorescence anisotropy. We recently estimated that more than one TDP-43 molecule may associate with the CLIP34 sequence (Grese et al., 2021). However, further studies are necessary to determine the actual number of TDP-43 molecules binding CLIP34. Due to this limitation, we analyzed the apparent IC_50_ (IC_50,*app*_) for the interaction between WT and mutant TDP-43 and CLIP34, as an estimate of the binding affinity. IC_50,*app*_ is the protein concentration in which 50% of the maximal anisotropy change is observed. Our results indicate that NTD mutations and ΔCR do not significantly affect CLIP34 binding (Table 1 and Supplementary Figure 3). As expected, we could not determine the binding of F4L for CLIP34 as these mutations greatly disrupt sequence-specific contacts (Buratti and Baralle, 2001; Lukavsky et al., 2013). Based on our results, we propose that the mutants bind CLIP34 as WT TDP-43 but fail to form condensates due to impaired self-assembly.

**TABLE 1:** Binding affinity of wild-type and mutant TDP-43 to CLIP34.

Protein	*IC*_50,*app*_ (μM)	IC*^Mut^*_50,*app*_ / IC*^WT^*_50,app_*
Wild-type (WT)	0.52 ± 0.08	1
S48A	0.66 ± 0.05	1.3
S48E	0.55 ± 0.04	1.1
E17R	0.43 ± 0.04	0.8
ΔCR (del. 316–346)	0.52 ± 0.08	1
ΔC (a.a. 1–269)	0.55 ± 0.08	1.1
S1 (a.a. 1–278)	1.1 ± 0.2	2
F4L	*Not determined*	

Values were obtained from binding experiments in Supplementary Figure 3. *Estimated loss (> 1) or gain (< 1) in binding affinity relative to WT.

To test the role of TDP-43 oligomerization and LLPS on autoregulation, we compared autoregulatory function in human cells expressing each of the LLPS-defective mutants. Stable HEK293 cells were generated expressing a single copy of the tagged TDP-43 transgene upon tetracycline induction, as previously described (Ayala et al., 2011). To determine autoregulatory activity of the mutants we measured protein and mRNA levels of endogenous TDP-43 in these cells at 72 h post transgene induction, which typically results in 70% reduction of endogenous TDP-43 levels in the case of WT (Figures 3D–H). In Figures 3F,H we report autoregulatory activity of the different mutants compared to WT at the protein and RNA levels calculated from the values of endogenous TDP-43 measured in Figures 3E,G. The ΔCR deletion showed a 50% decrease in autoregulatory activity at the protein and transcript level (Figures 3F,H), suggesting that LLPS is required for this function. We found a dramatic disruption of autoregulation in E17R cells at the protein and mRNA levels, resulting in >90% loss in activity compared to WT (Figures 3F,H). Similarly, S48E showed reduced autoregulatory function of approximately 50 and 70% compared to WT at protein and RNA levels, respectively. S48A function was not significantly different from WT, indicating that introduction of the negative charge at this site specifically disrupts autoregulation. In addition, these results suggest that the decrease in phase separation observed with S48A (Figures 3B,C) is not sufficient to significantly disrupt autoregulation in the cellular assay. Instead, our findings suggest that TDP-43 phosphorylation at Ser48 may be able to modulate autoregulatory activity and control TDP-43 levels in cells. These results indicate a crucial role of NTD-directed self-assembly in autoregulation, which is particularly highlighted by the degree of functional loss in E17R. This mutation was even more disruptive than the RNA-binding deficient mutant F4L (Ayala et al., 2011), here used as control (Figures 3E–H). Based on these observations, we propose that TDP-43 autoregulation requires TDP-43 self-assembly/oligomerization and condensate formation mediated by both N- and C-terminal domains. It is possible that these domains may also mediate heterotypic protein interactions that are important during autoregulation and this should be further investigated.

### TDP-43 Disease-Associated Variants Show Defects in CLIP34 LLPS and Autoregulatory Function

**FIGURE 4:**
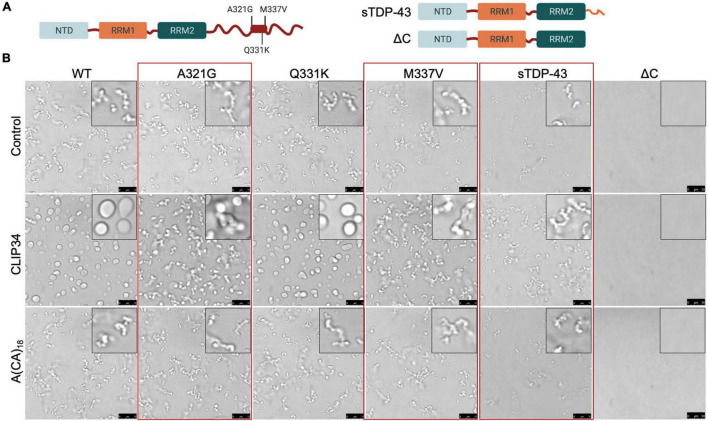
Specific ALS-associated TDP-43 mutations disrupt the liquid properties of Clip34-induced LLPS and alter autoregulation. **(A)** Single site amino acid substitutions associated with ALS A321G, Q331K and M337V, part of a highly conserved region and partially α-helical structure in the C-terminal domain (boxed region), are indicated. In addition, sTDP-43 incorporating a unique 18 a.a. C-terminal sequence is compared to the truncation mutant lacking the entire CTD, ΔC. **(B)** Phase separation of TDP-43 WT and mutants (4 μM) observed by brightfield microscopy in the presence of no RNA control, CLIP34 or A(CA)_18_ RNA (4 μM) at 150 mM NaCl, pH 7.5. Red boxes highlight variants that show differences from WT. Representative images of 3 biological replicates from 2 protein preparations. Scale bars, 10 μm.

We and others have shown that specific ALS-associated TDP-43 mutations alter the dynamic properties of TDP-43 condensates (Grese et al., 2021). Here, we investigated whether these TDP-43 mutations or variants linked to disease alter CLIP34-driven LLPS and consequently diminish autoregulatory function. We selected mutations positioned in the conserved region of the CTD (Figure 4A; Conicella et al., 2020). Phase separation of purified TDP-43 mutants A321G, Q331K and M337V was analyzed upon mixing with CLIP34 and compared to A(CA)_18_ or no RNA control (Figure 4B). We found that as in the case of WT TDP-43, CLIP34 promoted Q331K condensate liquidity, shown by increased fusion and coalescence into larger droplets. In contrast, A321G and M337V remained in clusters or chain-like structures in the presence of CLIP34, suggesting that these mutations retain their gel/solid properties and do not acquire liquid properties upon binding to this RNA molecule. Addition of A(CA)_18_ RNA did not significantly change the LLPS behavior of the mutants compared to control, as seen with WT TDP-43. Previous studies of the isolated CTD showed that these mutations disrupt LLPS through alterations in α-helix structure and helix-helix interactions (Conicella et al., 2016). Q331K and M337V similarly disrupt helix-helix contacts, while A321G shows helix-breaking behavior. However, we find that in the context of the full-length protein and RNA-driven condensation Q331K is distinct from M337V and A321G. The differences in the behavior of the mutants between these studies and the previous report (Conicella et al., 2016) may due to the use of the isolated CTD in previous work compared to the full-length protein in our studies, which specifically analyzed RNA binding-driven LLPS. The specific properties acquired by M337V and A321G, but not Q331K, should be further investigated as these may highlight important disease mechanisms. Next, we sought to determine whether a disease-associated short isoform of TDP-43 (sTDP-43), recently characterized by the Barmada group (Weskamp et al., 2020), alters protein condensation associated to autoregulation. Alternative splicing of *Tardbp* produces sTDP-43, which lacks the CTD and is replaced by a unique 18 amino acid long sequence (Figure 4A; Wang et al., 2004; D’Alton et al., 2015; Weskamp et al., 2020). This variant is toxic upon overexpression in neurons and is more highly expressed in ALS neurons and glia (Weskamp et al., 2020). We analyzed LLPS properties of sTDP-43 in the presence of CLIP34 and anticipated that loss of the entire CTD would strongly impair LLPS of sTDP-43, as in the case of ΔC (a.a.1-269) which lacks the entire CTD (Figure 4A) and prevented LLPS regardless of RNA presence, under the conditions tested (Figure 4B). In contrast, we found that the sTDP-43 variant spontaneously formed fibril-like structures (Figure 4B). Addition of CLIP34 did not promote liquid behavior, but increased abundance of the fibril complexes. The presence of A(CA)_18_ RNA did not significantly alter properties compared to control. The stark difference between sTDP-43, WT and ΔC suggests that the short peptide at the C terminus dramatically alters the conformation of the rest of the protein by conferring aggregation-prone properties. Alternatively, the short tail, unique to sTDP-43, may drive intermolecular interactions that promote fibrilization.

**FIGURE 5:**
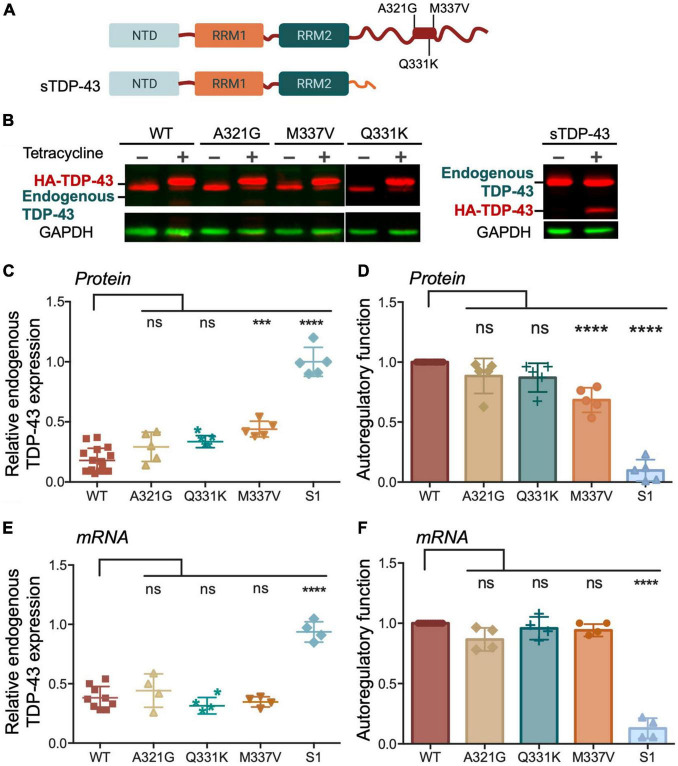
Disease associated TDP-43 variants suppress autoregulation. **(A)** Single site amino acid substitutions associated with ALS A321G, Q331K, M337V and sTDP-43 are depicted. **(B)** Representative immunoblots of HEK293 cell lines stably expressing HA-tagged TDP-43 upon tetracycline induction for 72 h. The transgene and endogenous TDP-43 signals appeared as indicated. Immunoblots were probed with TDP-43 antibody (red) and GAPDH as loading control (green). **(C)** Reduction of endogenous TDP-43 protein levels upon expression of the transgene quantified from panel **(B)**. Values calculated by the ratio of endogenous TDP-43 levels upon tetracycline induction, relative to non-induced conditions, both normalized to GAPDH. **(D)** Autoregulatory activity of the ALS mutants and sTDP-43, relative to WT, calculated from the values in panel **(C)** as endogenous TDP-43*^WT^*/endogenous TDP-43*^mutant^*. **(E,F)** Autoregulation in terms of *Tardbp* transcript levels measured by qPCR and quantified as in panels **(C,D)**. Mean and SD of 5 and 4 biological replicates for protein and RNA, respectively. Analyzed by one-way ANOVA, **(B)** [*F*(9,35) = 43.11, *P* < 0.0001], and **(C)** [*F*(9,34) = 82.45, *P* < 0.0001]. Dunnett’s multiple comparisons test was used to compare selected groups. ***P ≤ 0.001, ****P ≤ 0.0001. **(D)** Protein turnover estimated by measuring TDP-43 protein normalized to GAPDH at different time points post cycloheximide (CHX) treatment, as indicated. Cells expressing WT and M337V, as well as endogenous TDP-43, were treated with CHX (100 μg/mL) after 72 h of induced transgene expression. Mean and SD of ≥ 3 biological replicates.

We then investigated whether changes in condensate behavior seen with the ALS mutations affect autoregulation. We created stable HEK293 cell lines expressing a single copy of each mutant transgene upon tetracycline induction as for Figures 3D–H. To compare autoregulatory function between WT and mutants we quantified endogenous TDP-43 protein and mRNA levels in uninduced cells and after 72 h of induction (Figures 5A–F). We observed no significant differences in autoregulation in A321G and Q331K expressing cells relative to WT at protein and transcript levels (Figures 5B-F), suggesting that the mutants were similarly capable of autoregulation as WT TDP-43. Our results regarding the Q331K mutation are in contrast with observations using a knock-in mouse model expressing this mutant which results in altered *Tardbp* mRNA processing and increased TDP-43 protein and transcript levels (White et al., 2018). However, our observations are supported by similar behavior of Q331K dynamics in cells and autoregulatory activity compared to wild-type seen by others (Hallegger et al., 2021). The observed discrepancies between our cell-based model and the animal model may be caused by species or cell type-specific factors that influence TDP-43 condensation and/or autoregulation. As previously reported by Weskamp et al. (2020), we observed strong defects in sTDP-43 autoregulation using our stable cell line upon quantifying protein and mRNA levels of endogenous TDP-43 (Figures 5B–F). These results are consistent with the important role of the CTD in this function (Ayala et al., 2011). Because of the dramatic decrease in autoregulation associated with sTDP-43, we measured its binding affinity for CLIP34 RNA, comparing it to WT and ΔC (Table 1 and Supplementary Figure 3). While ΔC did not significantly change binding relative to WT, sTDP-43 caused a significant twofold increase in IC_50_, suggesting a moderate decrease in binding affinity of sTDP-43. The spontaneous fibrilization of sTDP-43 may account for this reduction in binding and should be further investigated. Nevertheless, based on previous studies, we predict that a decrease of binding affinity in this range is unlikely to alter cellular RNA processing function (Lukavsky et al., 2013). We also found that sTDP-43 was consistently expressed at lower protein levels compared to WT and the other mutants in the stable cell lines (Figure 5B). This may be caused by increased clearance of this isoform because of its propensity to aggregate/misfold, as seen in our assays (Figure 4B). Importantly, the decreased sTDP-43 protein levels may partly account for the reduction in its autoregulatory activity. Therefore, we propose that the extreme defects in autoregulation in the case of sTDP-43 arise from impaired LLPS, decreased protein expression, and impaired protein-protein interactions in the absence of the CTD. The behavior observed for sTDP-43 may partly explain the association of this isoform with neurotoxicity and TDP-43 pathology in neurons and in patients (Weskamp et al., 2020).

**FIGURE 6:**
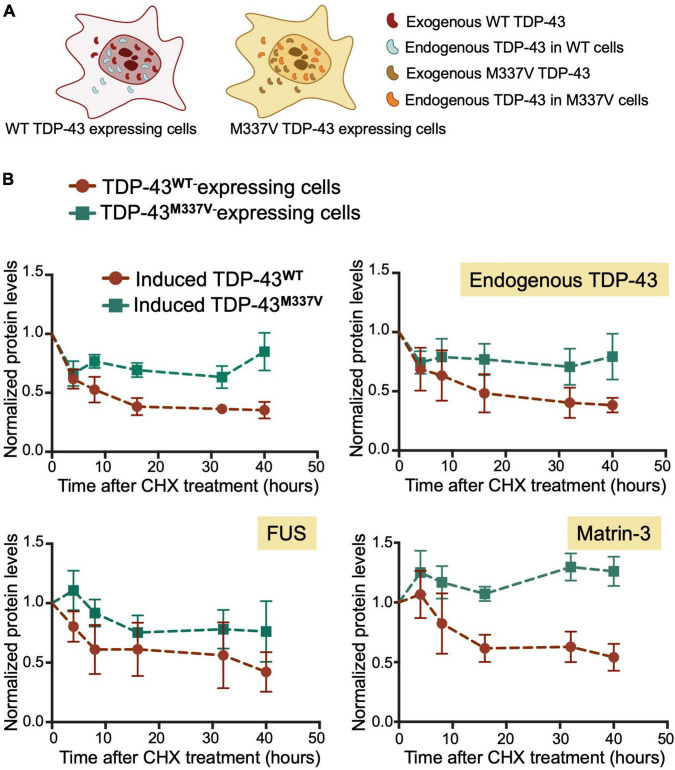
M337V suppresses protein clearance of TDP-43 and other RNA binding proteins linked to ALS. **(A)** Cells stably expressing WT (red) or M337V (cyan) TDP-43 upon tetracycline induction in the presence of endogenous TDP-43. **(B)** Turnover of induced and endogenous TDP-43, FUS and matrin-3 monitored by measuring protein levels normalized to GAPDH at different time points post-cycloheximide (CHX) treatment (100 μg/mL). Cells were treated with CHX after 72 h of induced transgene expression. Mean and SD of ≥ 3 biological replicates.

We found that M337V did not affect autoregulatory function when analyzing mRNA transcript levels (Figures 5E,F). These findings suggest that although M337V decreased the liquid properties of the condensates, these defects are not sufficient to significantly alter RNA processing in the case of autoregulatory function. Intriguingly, we found a 40% reduction in autoregulatory function when quantifying endogenous TDP-43 protein levels in M337V cells (Figures 5B–D). This difference derives from significantly greater accumulation of endogenous TDP-43 protein in M337V expressing cells, relative to WT (Figure 5C). To investigate the mechanism associated with this phenotype, we asked whether M337V increases protein aggregation in cells, as we previously observed using the purified protein (French et al., 2019). The M337V protein was found to be less soluble than the WT counterpart upon cell lysate fractionation into soluble and insoluble fractions (Supplementary Figures 4A–C). These results are in agreement with increased levels of soluble and insoluble TDP-43 in human induced pluripotent stem cells (iPSCs)-derived neurons expressing M337V (Bilican et al., 2012). Next, investigated whether M337V prevents the normal turnover and clearance of endogenous TDP-43 (Figure 6A). M337V-expressing cells were treated with cycloheximide (CHX), a translation inhibitor, and we measured the levels of induced, exogenous M337V and endogenous TDP-43 protein at different time points post-treatment. Cells expressing WT TDP-43 were similarly treated as control. Quantification of the protein levels for up to 40 h post-CHX treatment showed a stark increase in the half-life of induced M337V protein relative to induced WT protein (Figure 6B). Furthermore, the half-life of endogenous TDP-43 in M337V-expressing cells was also drastically increased relative to that measured in WT-expressing cells (Figure 6B). These results strongly suggest that M337V protein homeostasis is impaired and that these defects lead to aberrant accumulation of M337V that affects non-mutated TDP-43 in cells as well. We explored whether these defects may be reflected as differences in TDP-43 cellular localization. However, we did not observe significant changes in the nuclear-cytoplasmic localization of M337V compared to WT, or in the diffuse vs. focal organization of the transgene and endogenous proteins in the nucleus, based on immunofluorescence (Supplementary Figure 4D). Interestingly, we found that the levels of two other RNA binding proteins associated with ALS, FUS and matrin-3, also showed abnormal accumulation post-CHX treatment in M337V, compared to WT-expressing cells (Figure 6B). The stark differences in the proteostasis of M337V may be caused by defects in the loss of liquid properties unique to this mutation as seen by our *in vitro* condensation assays. These intriguing findings also suggest that the defects caused by M337V alter general pathways of cellular proteostasis, affecting aggregation-prone proteins that are relevant to disease.

## Discussion

The autoregulatory activity of TDP-43 is central to its function in RNA processing as even moderate changes in TDP-43 levels may significantly alter gene expression. The importance of autoregulation for TDP-43 metabolism is further highlighted by two lines of evidence. First, *in vivo* models of TDP-43 pathology and patient-derived tissue show aberrantly increased levels of TDP-43 expression (Gitcho et al., 2009; White et al., 2018; Liu et al., 2019). Second, defects in autoregulation cause TDP-43-associated neurotoxicity (Tatom et al., 2009; Barmada et al., 2010; Wils et al., 2010; Xu et al., 2010; Estes et al., 2011; Uchida et al., 2012; Diaper et al., 2013; White et al., 2018). These observations strongly indicate that impaired autoregulation may result in elevated protein levels, increasing the aggregation propensity of TDP-43. Here, we show that this essential TDP-43 function is mediated by TDP-43 condensation into ribonucleoprotein complexes through a mechanism implicating specific RNA binding and protein domain interactions. We show that TDP-43 binding to CLIP34 RNA specifically, whose sequence forms part of the TDP-43 binding region mediating autoregulation, increases the liquid properties of TDP-43 condensates. Importantly, we find that TDP-43 ALS-associated mutations and the short variant isoform sTDP-43 affect different interactions involved in this process. In particular, our studies provide insight into possible pathogenic mechanisms associated with the M337V mutant and the short TDP-43 isoform associated with neurotoxicity in ALS. These findings provide mechanistic insight of an important process regulating TDP-43 homeostasis and may help in the design of new therapeutic strategies to control TDP-43 function and solubility.

Based on our results, TDP-43 autoregulation requires TDP-43-self-assembly and condensate formation. We find that disruption of TDP-43 LLPS activity through targeted mutations strongly impairs autoregulatory activity (Figure 3). These results are supported by studies from the Ule lab published while this manuscript was in preparation (Hallegger et al., 2021). Hallegger et al. (2021) showed that condensation activity is correlated with autoregulatory function by disrupting the conserved α-helical region in the LCD. Here, we show that in addition to interactions mediated by the LCD, self-assembly through the N-terminal domain of TDP-43 is essential for autoregulation (Figure 3). This mechanism of oligomerization is necessary for TDP-43-mediated splicing regulation of numerous targets and regulation of R-loop metabolism, according to our previous data and others (Afroz et al., 2017; Wang et al., 2018; Wood et al., 2020), suggesting that these processes and autoregulation share common mechanisms. Our results showing that autoregulation-linked condensation requires RNA binding as well as NTD and interactions is consistent with the pivotal role of multivalent interactions in mediating condensate networks. We also note that, at this time, we may not rule out that the N and C-terminal regions are required for oligomerization or interactions with protein partners, in the absence of phase separation. While this possibility needs to be investigated, we provide strong evidence for an important role of TDP-43 condensation during autoregulation. We showed that CLIP34 specifically and potently induces liquid-liquid phase separation of purified TDP-43 as well as in the presence of cellular lysate and promotes liquid properties of TDP-43 droplets. In addition, our work shows inhibition of autoregulation following the introduction of mutations that inhibit LLPS. Interestingly, our findings suggest that TDP-43 autoregulation may be controlled by phosphorylation at residue S48 in the N-terminal domain, which strongly inhibits self-assembly and phase separation (Figure 3; Wang et al., 2018). The cellular pathway controlling S48 phosphorylation is unknown, yet our data suggests that it may be a powerful modulator of TDP-43 function and proteostasis.

To explore possible links between the condensation-autoregulation process and TDP-43-associated disease, we investigated the function of TDP-43 mutations causative of ALS. Among the disease mutations within the CTD conserved region, we found that A321G and M337V were able to form condensates on their own, similar to WT. However, CLIP34 RNA did not confer liquid properties to the condensates formed by these mutants, in contrast to WT and the other mutations tested (Figure 4). These defects were consistent with our recent observations that GU-repeat RNA binding is not capable of increasing the liquid properties to A321G and M337V condensates, in contrast to its effect on WT TDP-43 (Grese et al., 2021). The altered behavior of A321G and M337V suggests less dynamic assemblies compared to WT, which may result in accelerated conversion into fibrils or aggregates. This model is consistent with our previous findings that M337V accelerates aggregate formation and increases aggregate seeding capacity in cells (French et al., 2019). In addition, aberrant M337V condensate behavior is supported by defects in stress granule function observed in a previous M337V mouse model (Gordon et al., 2019). However, despite the observed loss in liquid properties, A321G and M337V mutants did not significantly decrease autoregulatory function at the transcript level, compared to WT (Figure 5). This is in contrast with mutations that greatly decreased or even blocked LLPS entirely, such as ΔCR and NTD mutations (Figure 3). We propose that the ability of A321G and M337V to form condensates, albeit of decreased liquidity, is sufficient for autoregulation in cells where additional protein interactions (e.g., chaperones) may counteract defects in A321G and M337V complexes. It is also possible that additional regulatory factors in cells, such as 3′UTR RNA sequences flanking CLIP34 in the entire TDPBR are able to rescue the defects observed with the purified A321G and M337V proteins.

Our present studies led to intriguing observations of disease-associated factors disrupting normal TDP-43 homeostasis. We found significantly increased accumulation of endogenous TDP-43 protein in M337V expressing cells, compared to wild-type (Figures 5B–D). These results are consistent with previous reports of neurons derived from M337V carrier patients, which show decreased survival and higher levels of total TDP-43 compared to control (Bilican et al., 2012). We also find that elevated endogenous TDP-43 protein levels were caused by a dramatic decrease in its clearance upon co-expression of M337V (Figure 6). The M337V protein also shows greatly reduced turnover compared to WT, as was previously reported in a different human cell line (Yin et al., 2021). Of note, the WT and M337V proteins were expressed in isogenic cell lines in our studies, suggesting that M337V homeostasis is intrinsically different from WT. Based on these results, we speculate that structural and functional defects in M337V disrupt the clearance of non-mutated TDP-43 in the affected cells, having the overall effect of increasing TDP-43 levels. Moreover, our observations that FUS and matrin-3 proteins also showed abnormal accumulation in M337V cells suggest strong defects in cellular protein homeostasis. Based on the accelerated maturation of M337V condensates, we speculate that M337V sequesters TDP-43, and perhaps other related proteins (e.g., FUS, matrin-3), into insoluble aggregates. It is also possible that the decreased solubility of M337V (Supplementary Figure 4) reduces the capacity of cellular protein clearance through a yet uninvestigated mechanism. These findings are relevant to patients carrying the M337V mutation as heterozygous, whereby expression of the M337V protein also affects the homeostasis of the WT allele as well as other proteins.

Our findings show that TDP-43 binding to CLIP34, specifically modulates liquid-liquid phase separation properties of TDP-43 (Figure 1). We previously showed evidence that TDP-43 LLPS is upregulated with increasing number of available RNA binding sites. According to band shift analyses, we estimated that 3-4 TDP-43 molecules bind to CLIP34 (Grese et al., 2021). The increased valency of the RNA-bound complex is likely to increase TDP-43 phase separation. Importantly, this sequence-specific RNA binding activity is also demonstrated in a cellular environment using human cell lysate (Figure 2). These results are consistent with our recent findings that interactions with specific RNA sequences promote TDP-43 condensation, and at the same time, increase the liquidity of phase separation (Grese et al., 2021). Additionally, Mann et al. (2019) previously showed that CLIP34 RNA decreases TDP-43-mediated neurotoxicity. Collectively, this evidence indicates that specific TDP-43-RNA interactions are important to prevent TDP-43 aggregation in the condensed state, during the processing of specific targets.

## Data Availability Statement

The original contributions presented in the study are included in the article/Supplementary Material, further inquiries can be directed to the corresponding author/s.

## Author Contributions

LK, ZG, AB, and YA designed the study. ZG and LM generated the recombinant protein and performed the phase separation analysis. LK carried out the cell biology experiments and analyses. AB measured the RNA binding affinity. TH participated in the design, analysis and interpretation of the RNA binding assays. LM generated the DNA constructs and mutagenesis. YA wrote the manuscript. LK, ZG, AB, LM, and YA reviewed and edited the manuscript. All authors contributed to the article and approved the submitted version.

## Conflict of Interest

The authors declare that the research was conducted in the absence of any commercial or financial relationships that could be construed as a potential conflict of interest.

## Publisher’s Note

All claims expressed in this article are solely those of the authors and do not necessarily represent those of their affiliated organizations, or those of the publisher, the editors and the reviewers. Any product that may be evaluated in this article, or claim that may be made by its manufacturer, is not guaranteed or endorsed by the publisher.

## Supplementary Material

Supplementary Figure 1 CLIP34 RNA specifically increases the liquid properties of TDP-43 condensates. The size of TDP-43 condensates in the presence and absence of RNA was quantified as an estimation of differences in their liquid properties. The area of condensates composed of purified TDP-43 control, in the presence of CLIP34 or A(CA)_18_ RNA shown in Figure 1C was calculated by ImageJ. Mean and SD of > 600 condensates from three biological replicates using two different protein preparations. Analyzed by one-way ANOVA [*F*(2,1948) = 353.5, *P* < 0.0001]. Dunnett’s multiple comparisons test was used to compare groups to WT. *****P* ≤ 0.0001. ns, no significance.

Supplementary Figure 2 CLIP34 RNA promotes TDP-43 condensation in a cellular lysate at 150 mM NaCl. **(A)** Cell lysates were prepared from HEK 293 cells stably expressing a single mGFP-tagged copy of wild-type (WT) TDP-43. 4% of this lysate was added to recombinant TDP-43 (5.3 μM) mixed with CLIP34 (7.6 μM), A(CA)_18_ (7.6 μM), or no RNA control at 150 mM NaCl, pH 7.5. Representative images of 3 biological replicates from 2 protein preparations. Scale bars, 10 μm.

Supplementary Figure 3 TDP-43-CLIP43 binding assays. **(A–G)** Representative binding analysis for WT and mutants measured by fluorescence anisotropy of FITC-labeled CLIP34 RNA (100 nM) titrated with increasing TDP-43 WT and mutants (0–2 μM). Each point is the average of > 3 biological replicates (SEM). The calculated apparent IC_50_ (*IC*_50,*app*_) is the protein concentration in which 50% of the maximal anisotropy change is observed. The values obtained are summarized in Table 1. **(H)** ANOVA analysis of the IC_50_ values comparing WT and mutant TDP-43. ****P* ≤ 0.001; ns, no significance.

Supplementary Figure 4 The ALS-associated M337V mutation decreases solubility and affects the solubility of co-expressed endogenous TDP-43. **(A)** TDP-43 solubility measured in HEK293 cells stably expressing a single copy of WT or M337V upon tetracycline induction. **(B)** Lysates of cells induced for 72 h were fractionated into RIPA and Urea-soluble fractions. The levels of transgene and endogenous TDP-43 were measured by immunoblotting (representative blots shown). Blots were probed with TDP-43 and GAPDH as loading control. High exposure of the immunoblot is included to compare levels in the Urea fraction. **(C)** The proportion of soluble WT and M337V transgenes is plotted in addition to that of endogenous TDP-43 from WT and M337V-expressing cell lines. Mean and SD of ≥ 3 biological replicates. **(D)** Representative immunofluorescence of HA-TDP-43 WT and M337V-expressing cells showing the predominantly nuclear detection of endogenous and HA-tagged TDP-43, *n* = 3 biological replicates. Scale bars, 10 μm.

Supplementary Figure 5 M337V expression decreases the clearance of RNA binding proteins linked to ALS. **(A,B)** Representative immunoblots of HEK293 cells stably expressing HA-tagged WT or M337V TDP-43 for 72 h upon tetracycline induction. Cells were then treated with cycloheximide (CHX, 100 μg/mL) and samples were taken at different time points post-CHX treatment, as indicated. Membranes were probed with TDP-43, Matrin-3, and FUS antibodies. GAPDH was used as a loading control.

Supplementary Table 1 List and sequence of DNA oligonucleotides used for cloning and mutagenesis.

Supplementary Movies 1–3 Specific RNA promotes liquid properties of TDP-43 condensates. TDP-43 (4 μM) in no-RNA conditions (1) or mixed with CLIP34 (4 μM) (2) or A(CA)_18_ (4 μM) (3) at 150 mM NaCl. Related to Figure 1. Scale bar, 25 μm.
