# Supplementary material for: TDP-43 Oligomerization and Phase Separation Properties Are Necessary for Autoregulation
Source: Front Neurosci. 2022 Apr 14;16:818655. doi: 10.3389/fnins.2022.818655 (PMC9048411; doi:10.3389/fnins.2022.818655)
Supplement: Supplementary Figure 1 — CLIP34 RNA specifically increases the liquid properties of TDP-43 condensates. The size of TDP-43 condensates in the presence and absence of RNA was quantified as an estimation of differences in their liquid properties. The area of condensates composed of purified TDP-43 control, in the presence of CLIP34 or A(CA)18 RNA shown in Figure 1C was calculated by ImageJ. Mean and SD of > 600 condensates from three biological replicates using two different protein preparations. Analyzed by one-way ANOVA [F(2,1948) = 353.5, P < 0.0001]. Dunnett’s multiple comparisons test was used to compare groups to WT. ****P ≤ 0.0001. ns, no significance. [file Data_Sheet_1.pdf]

Supplementary Fig. 1

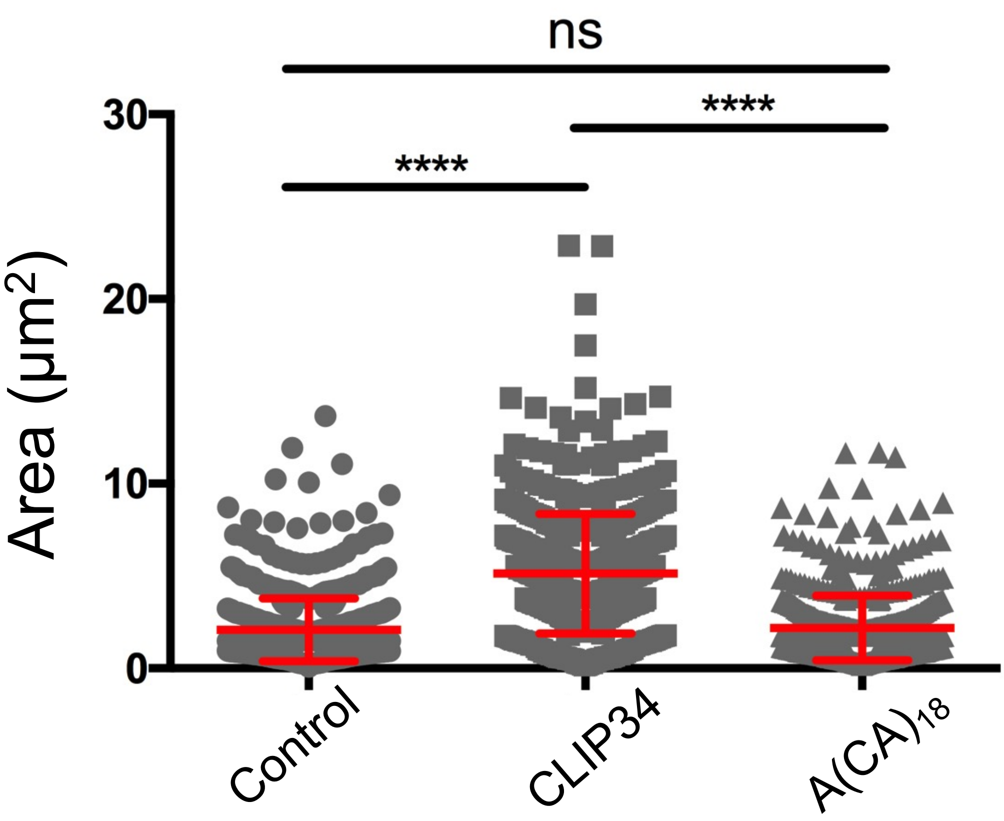

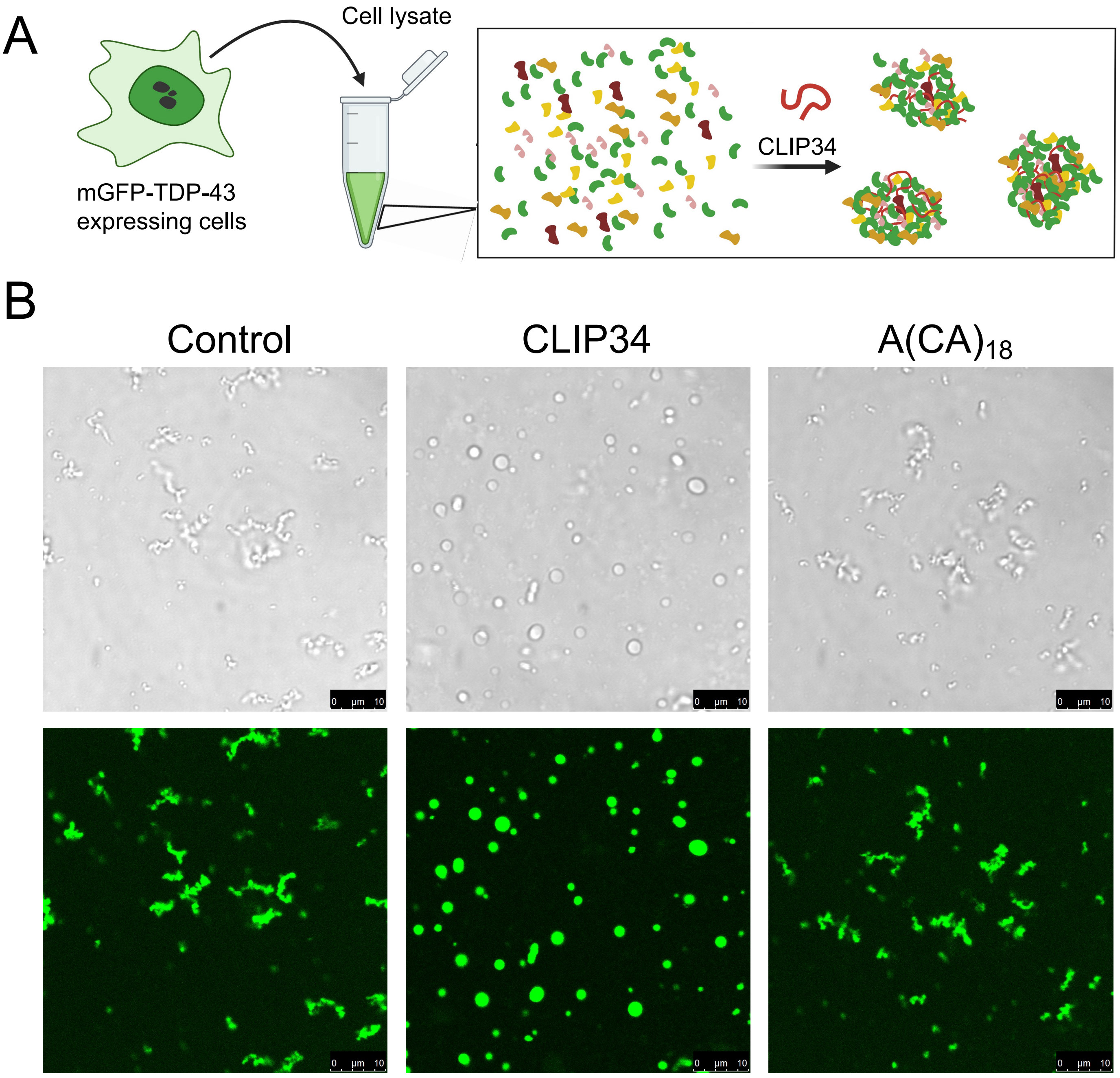
A
Cell lysate
mGFP-TDP-43
expressing cells
CLIP34
B
Control
CLIP34
A(CA)18
0 µm 10

Supplementary Fig. 3

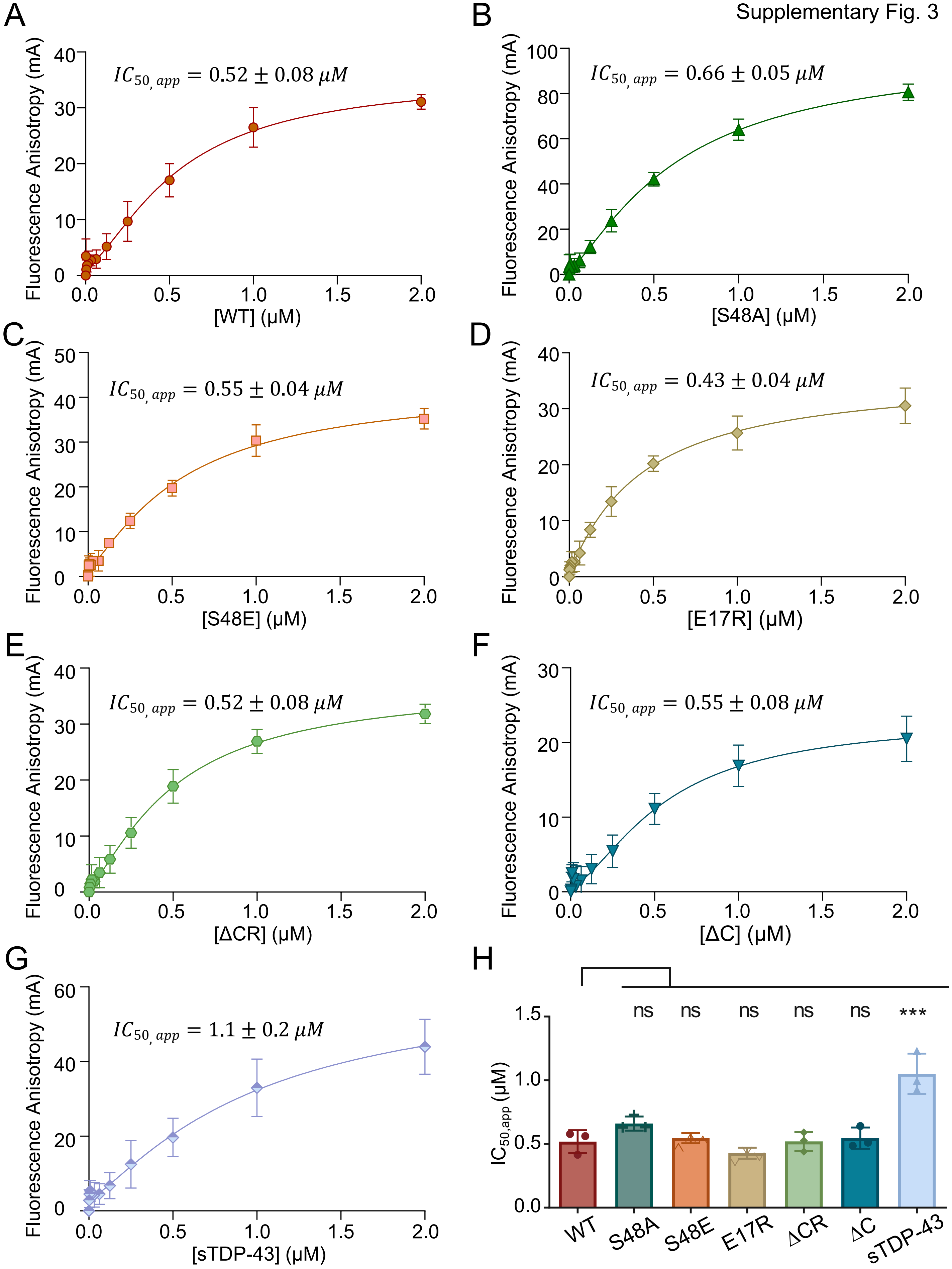

Supplementary Fig 4

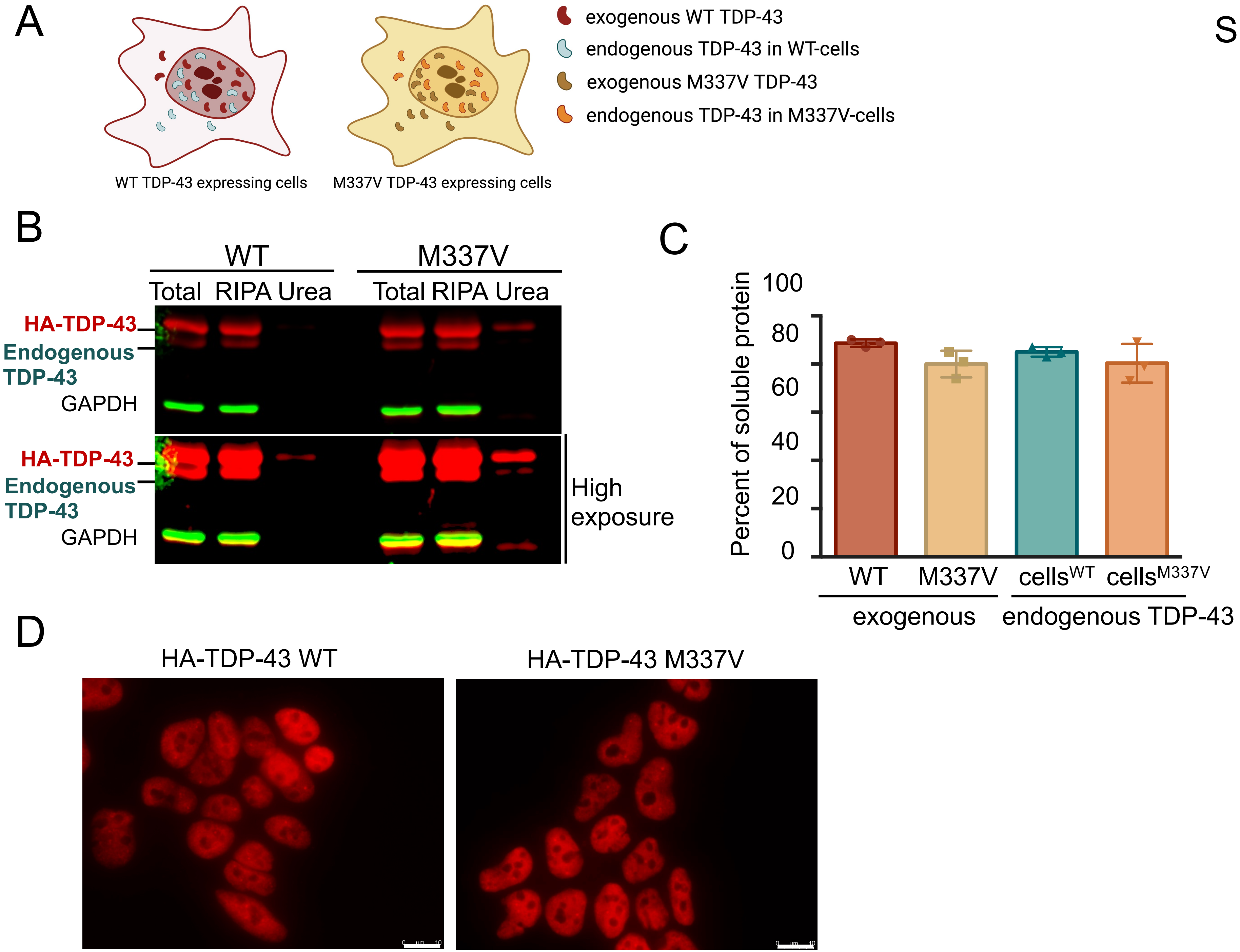

Supplementary Fig 5

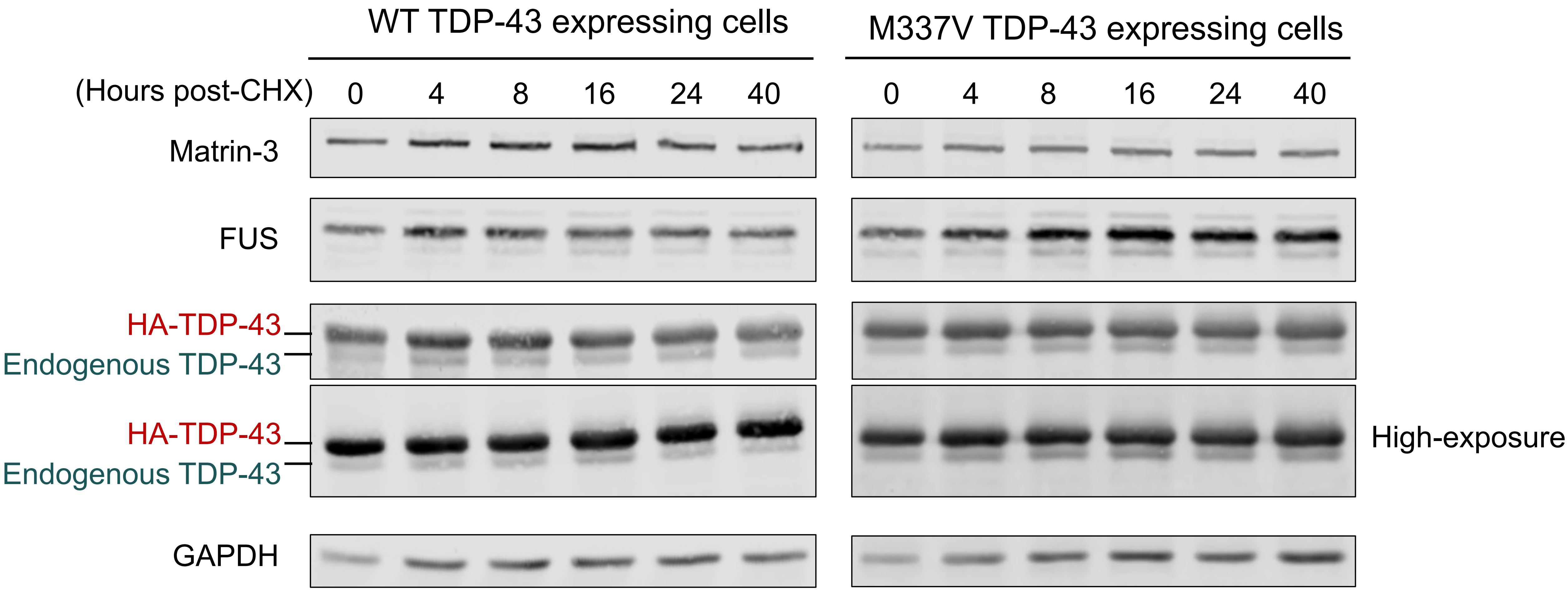

Supplementary Table 1
List and sequence of DNA oligonucleotides used for cloning and mutagenesis

TDP-43 Mutant		Oligonucleotide sequence (5'-3')
S48A	FW<br>RV	CGCTACAGGAATCCAGTGGCTCAGTGTATGAGAGGTGTC<br>GACACCTCTCATACACTGAGCCACTGGATTCCTGTAGCG
S48E	FW<br>RV	CGCTACAGGAATCCAGTGGAACAGTGTATGAGAGGTGTC<br>GACACCTCTCATACACTGTCCCACTGGATTCCTGTAGCG
E17R	FW<br>RV	AGAACGATGAGCCCATTCGAATACCATCGGAAGACGATG<br>CATCGTCTTCCGATGGTATTCGAATGGGCTCATCGTTCT
F147/149L	FW<br>RV	GACTGGTCATTCAAAGGGGCTTGGCCTTGTTCGTTTTACGGAATATG<br>CATATTCCGTAAAACGAACAAGGCCAAGCCCCTTTGAATGACCAGTC
F229/231L	FW<br>RV	CCAAGCCATTCAGGGCCCTTGCCCTTGTTACATTTGCAGATGATC<br>GATCATCTGCAAATGTAACAAGGGCAAGGGCCCTGAATGGCTTGG
sTDP-43	RV	GGCAGCGGCCGCTTACAGCACTACTTTCAATGAAGTGCTTCTTCCATAAA<br>CATT TGAAATGAGATGAACACCAAATCTTCCACTTCTTTC
A321G	FW<br>RV	TTTGGTGCTTCAGCATTAATCCAGGCATGATGGCTGCCGCCCAGGCAG<br>CTGCCTGGGCGGCAGCCATCATGCCTGGATTAATGCTGAACGCACCAAA
Q331K	FW<br>RV	GCCGCCCAGGCAGCACTAAAGAGCAGTTGGGGTATGATG<br>CATCATACCCCAACTGCTCTTTAGTGCTGCCTGGGCGGC
M337V	FW<br>RV	CAGAGCAGTTGGGGTATGGTGGGCATGTTAGCCAGCCAG<br>CTGGCTGGCTAACATGCCCACCATACCCCAACTGCTCTG

Supplementary Table 2
List and sequence of DNA oligonucleotides used for quantitative real-time PCR

Primer name	Oligonucleotide sequence (5'-3')
GAPDH FW	AAGGTGAAGGTCGGAGTCAA
GAPDH RV	AATGAAGGGGTCATTGATGG
RThV1x6FWc	TGAACTGCTGTTTGCCTGATTG
RThV1x6RVc	GTGGGGTTCAAATTAAAACAAGGG

Supplementary Table 3
List and sequence of **RNA** oligonucleotides used for fluorescence anisotropy and phase separation

Name	Oligonucleotide sequence (5'-3')
CLIP34	GAGAGAGCGCGUGCAGAGACUUGGUGGUGCAUAA
A(CA)18	ACACACACACACACACACACACACACACACACACACA
